# Safety and use of pulmonary function tests: a retrospective study from a single center over seven years’ clinical practice

**DOI:** 10.1186/s12890-019-1019-z

**Published:** 2019-12-21

**Authors:** Fei Li, Zhi-wen Huang, Xiao-fei Wang, Hui-wen Xu, Hua Yu, Yan-bin Chen, Jian-an Huang, Jia-jia Wang, Wei Lei

**Affiliations:** 1 Department of Pulmonary and Critical Care Medicine, The First Affiliated Hospital of Soochow University, Suzhou, 215006 Jiangsu China; 2 Department of Respiratory Medicine, Affiliated Renhe Hospital of China Three Gorges University, Yichang, 443001 Hubei China; 3 Department of Surgery, Cancer Control, University of Rochester School of Medicine and Dentistry, Rochester, NY 14642 USA; 4 Department of Respiratory Medicine, First People’s Hospital of Fuzhou, Fuzhou, 344000 Jiangxi China

**Keywords:** Pulmonary function tests, Adverse events, Cost-effectiveness, Patient safety

## Abstract

**Background:**

To promote the utilization of pulmonary function tests (PFT) through analyzing the data of PFT during the past seven years in one large teaching hospital in China.

**Methods:**

Through a retrospective analysis, the allocation of full-time staff in PFT room, the demographic characteristics of patients, cost-effectiveness of PFT, positive rate and failure rate of PFT, adverse events were analyzed.

**Results:**

1) From 2012 to 2018, the numbers of PFT showed the trend of escalation year by year. The proportion of patients receiving PFT rose from 29.0/10,000 in 2012 to 34.7/10,000 in 2018. The best allocation of PFT room was 20–25/ person / day. 2) The number of PFT provided by Department of Pulmonary and Critical Care Medicine (PCCM) accounted for 97.2, 97.1, 97.3, 97.8, 97.8, 98.0, and 98.2% of the total cases of outpatient PFT in the same year. The top three departments in the inpatient department were Department of Thoracic Surgery, Department of General Surgery, and Department of Urinary Surgery, the total cases of PFT in these three departments accounted for 65.1, 64.4, 62.1, 63.5, 62.4, 65.3 and 69.1% of the total cases of inpatient PFT in the same year. 3) Data from 2018 showed that the revenue from PFT was about 3.7 million Chinese Yuan, and that the salary of personnel and expenditure on machine maintenance and wear were about 800,000 Chinese Yuan. 4) 58.2% of the patients who had undergone PFT had ventilatory dysfunction. 5) The average failure rate of PFT in the past seven years was 1.91%. 6) The main adverse events of PFT examination were dizziness, amaurosis, limb numbness, lip numbness and falls. The incidence rates were 0.49, 0.42, 0.41, 0.39, 0.44, 0.48, and 0.45% respectively, with an average of 0.44%.

**Conclusions:**

The number of PFT showed an upward trend in the past seven years, and the optimal staffing of PFT room was 20–25 cases per person per day. The positive rate of pulmonary dysfunction was 58.2%. The failure rate of PFT and the incidence of adverse events were very low, suggesting it is a simple and safe clinical examination. It’s worthy of further popularization and promotion.

## Background

Pulmonary function tests (PFT) is an important diagnostic tool for epidemiology and clinical evaluation of respiratory diseases. It is also an important method for preoperative evaluation of pulmonary function. It is of great value in early diagnosis, severity classification, disease progression and evaluation of curative effect of respiratory diseases [1–4].

Although people pay more attention to medical treatment and health care, the utilization of PFT is still unsatisfactory, far less common than the measurement of blood pressure and blood sugar, especially in second-class hospital. It’s mainly because of the expensive of PFT equipment, the high cost of PFT, potential adverse events for certain patients (especially for elderly patients), high requirement of operators’ experience and patients’ cooperation as well as lack of publicity of PFT [5].

We conducted a comprehensive analysis of the data of the PFT carried out in the past seven years, the staffing of the PFT room, the cost-effectiveness of the PFT, the failure rate and the adverse events during the test, and the positive rate of pulmonary dysfunction in this study. We aim to provide some reference value for the further promotion of PFT.

## Methods

### Data collection

Staffing and equipment in the PFT room of our hospital (a large teaching hospital in China) and the test results in the past seven years (January 2012– December 2018) were collected. All information related to the PFT was analyzed statistically, which included demographic information of patients, the numbers of PFT, the distribution of patients received PFT, cost-effectiveness of the test, the positive rate of pulmonary dysfunction, the failure rate and the adverse events of PFT.

All patients who were prescribed PFT obtained their consents. The patients who finished the examination would be asked the following two questions: 1) Did you have any discomfort during PFT? 2) What kind of discomfort did you have during PFT? The adverse events would be documented. If the patients didn’t finished the examination, the causes of failure would be recorded.

### Statistical analysis

All statistical analyses were performed using the SPSS 22.0 statistical software (IBM Corp., New York, NY). Counting data were represented by numbers and described as a constituent ratio.

## Results

### The number of PFT showed the trend of escalation in the past seven years

#### In the past seven years, the majority patients were elderly

For the past seven years, the number of PFT was 5754, 5929, 7128, 8775, 10,659, 13,121, and 15,825 respectively. It increased by 20% each year. The majority patients were male and it’s 1.31–1.50 times than females patients. We made an analysis of the proportion of patients receiving PFT visited hospital in the last seven years, the mean proportion was 30.8 cases/ 10,000 people. It increased from 29.0 cases/ 10,000 people in 2012 to 34.7 cases/ 10,000 people in 2018.

**Fig. 1:**
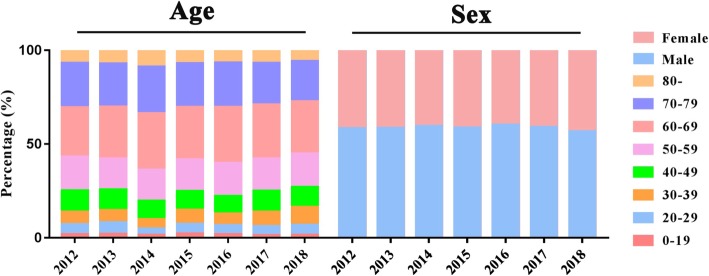
Demographic characteristics of patients in recent seven years

The proportion of PFT for people under 40 years of age in recent seven years was 13.9, 14.6, 9.8, 14.8, 13.0, 13.8, and 16.3% respectively. In the past seven years, 56.7, 57.9, 63.8, 58.3, 60.0, 57.8, and 55.1% of the elderly (60 years and older) were carried out for PFT (Fig. 1).

#### The peak period of PFT every year was around March and November

**Fig. 2:**
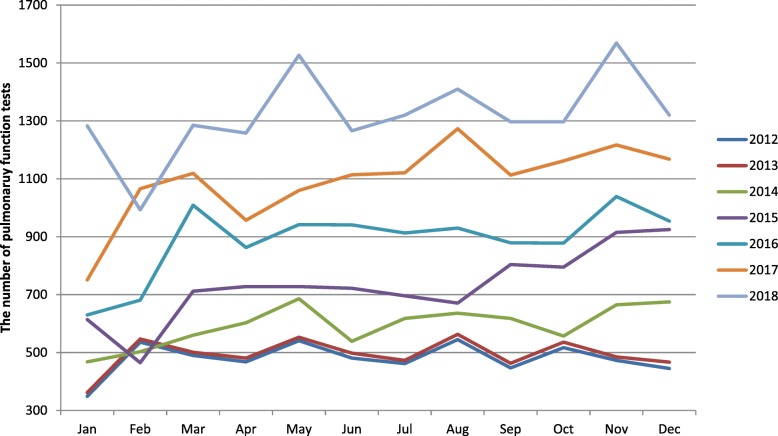
Monthly changes of PFT in recent 7 years. PFT, pulmonary function tests

The analysis showed that the annual PFT for the last seven years peaked around in March and November (Fig. 2), which was related to the high incidence of respiratory diseases in winter and spring. In addition, the number of PFT in the same period each year was basically on the rise, suggesting the importance of PFT had been increasingly emphasized.

### The best allocation of PFT was 20–25 cases/ person/ day

With the increasing workload of PFT year by year, the fully trained staff in PFT room increased from 1 person to 4 persons. The staff also needed to have training courses related to PFT, to improve their professional skills. By analyzing the time consumed in PFT (pulmonary ventilation function (i.e. spirometry and static volumes measurements), 10 min; pulmonary ventilation function + bronchial dilation test, 30 min; pulmonary ventilation function + bronchial provocation test, 60 min; pulmonary ventilation function + diffuse function (i.e. DLCO measurement, generally used the method of single-breath determination of carbon monoxide uptake in the lung), 15 min) and the workload of PFT room, the best allocation of PFT was 20–25 cases / person / day.

### Outpatients who received PFT were mainly from Department of Pulmonary and Critical Care Medicine, and inpatients who received PFT were mainly from Department of Thoracic Surgery

#### The distribution of outpatients received PFT

Patients who received PFT were mainly from outpatient and inpatient (Table 1). Over the past seven years, outpatient and inpatient departments had shown a steady increase in the number of PFT. The majority of these patients were from Department of Pulmonary and Critical Care Medicine (PCCM), Department of Cardiovascular Medicine (DCM), Department of Hematology (DH), Department of General Surgery (DGS), and Department of Physical Examination (DPE). The number of PFT in PCCM ranked first, accounting for 97.2, 97.1, 97.3, 97.8, 97.8, 98.6 and 98.2% respectively.

**Table 1:** The distribution of patients received PFT in recent seven years

	2012	2013	2014	2015	2016	2017	2018
Outpatient (%)	2186 (38.0)	2241 (37.8)	2676 (37.5)	3016 (34.4)	3739 (35.1)	4926 (37.5)	6511 (41.1)
Inpatient (%)	3568 (62.0)	3688 (62.2)	4452 (62.5)	5759 (65.6)	6920 (64.9)	8195 (62.5)	9314 (58.9)
Total number (%)	5754 (100)	5929 (100)	7128 (100)	8775 (100)	10,659 (100)	13,121 (100)	15,825 (100)

#### The distribution of inpatients received PFT

**Fig. 3:**
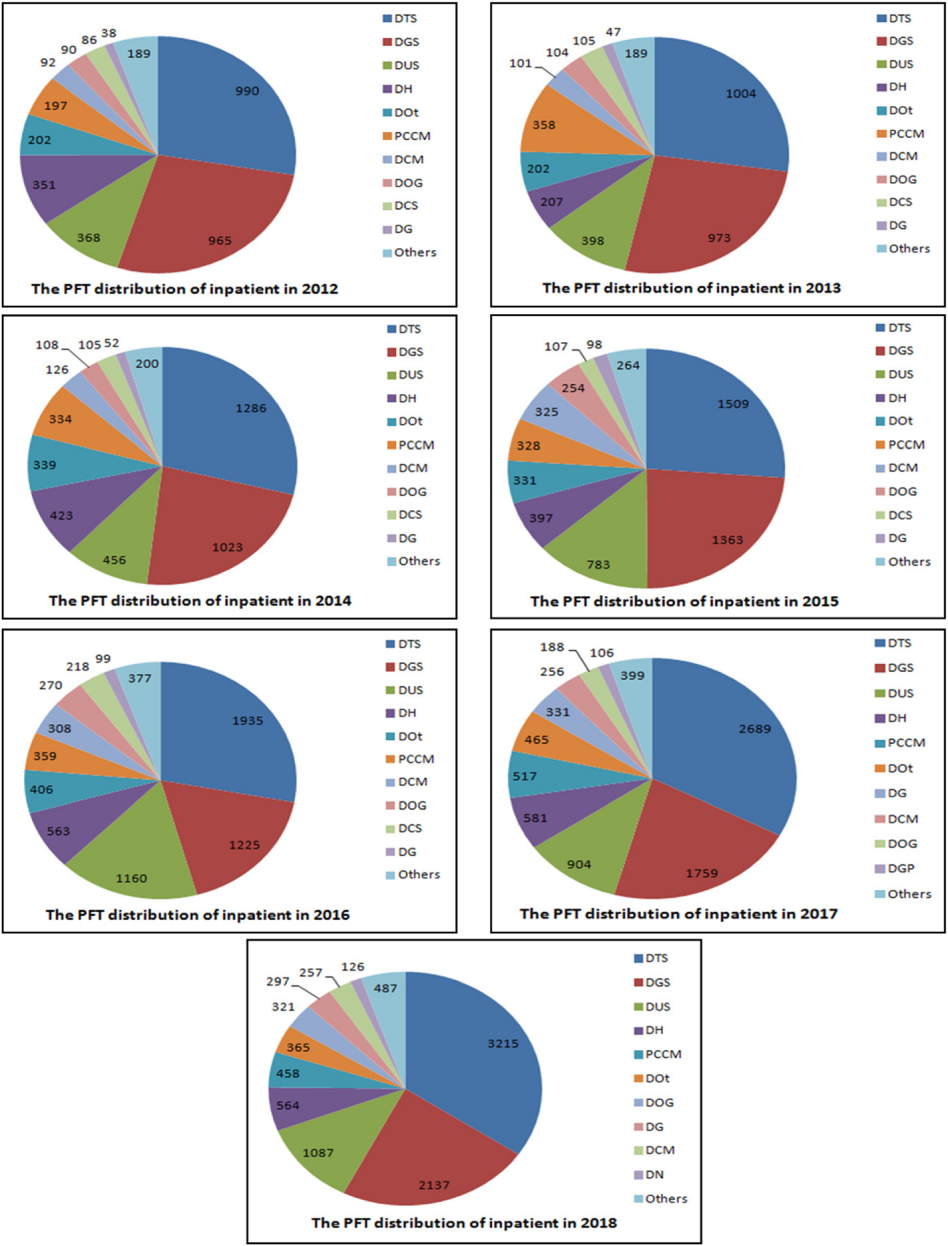
The PFT distribution of inpatient in recent 7 years. PFT, pulmonary function tests; PCCM, Department of Pulmonary and Critical Care Medicine; DTS, Department of Thoracic Surgery; DGS, Department of General Surgery; DUS, Department of Urinary Surgery; DH, Department of Hematology; DOt, Department of Otorhinolaryngology; DCM, Department of Cardiovascular Medicine; DOG, Department of Obstetrics and Gynaecology; DCS, Department of Cardiovascular Surgery; DG, Department of Gastroenterology; DOr, Department of Orthopedics. DGP, Department of General Practice; DN, Department of Neurosurgery

By analyzing the distribution of inpatient, the top 10 from 26 departments were of the same situation (Fig. 3). The top three departments were Department of Thoracic Surgery (DTS), Department of General Surgery (DGS), and Department of Urinary Surgery (DUS). They accounted for 65.1, 64.4, 62.1, 63.5, 62.4, 65.3, and 69.1% of the total cases, which also showed the importance of preoperative risk assessment of thoracoabdominal surgery.

### High economic benefits with great social value

#### The economic benefits of PFT

The data of 2018 was analyzed statistically. In 2018, PFT was performed in 15,825 cases, and the average cost of each test was 235 Chinese Yuan. The total revenue of PFT was about 3.7 million Chinese Yuan. Excluding the cost of personnel salary, machine maintenance and wear which was about 800,000 Chinese Yuan, the economic interest was still considerable.

#### The social value of PFT

There were 4 diagnostic results of PFT [6, 7]: normal, obstructive abnormalities, mixed abnormalities, restrictive abnormalities (Table 2). In the past seven years, the proportion of patients with different severity of ventilatory defects were 49.9, 58.9, 63.2, 58.8, 57.0, 58.7 and 58.6% respectively. On the average, there was 58.2% of the patients found to have ventilatory defects, which indicated the vital social value and relevant clinical value of PFT.

**Table 2:** Positive screening rate of ventilatory defects

	2012	2013	2014	2015	2016	2017	2018
Total (%)	5629 (100)	5761 (100)	6972 (100)	8589 (100)	10,501 (100)	12,912 (100)	15,547 (100)
Normal (%)	2822 (50.1)	2370 (41.1)	2567 (36.8)	3535 (41.2)	4518 (43.0)	5335 (41.3)	6433 (41.4)
Obstructive abnormalities (%)	2083 (37.0)	2554 (44.3)	3267 (46.9)	3624 (42.2)	4336 (41.3)	5452 (42.2)	6666 (42.9)
Mixed abnormalities (%)	457 (8.1)	492 (8.5)	691 (9.9)	883 (10.3)	975 (9.3)	1266 (9.8)	1489 (9.6)
Restrictive abnormalities (%)	267 (4.7)	345 (6.0)	447 (6.4)	547 (6.4)	672 (6.4)	859 (6.7)	959 (6.2)

### The failure rate of PFT was only 1.91%

The failure rates of PFT in the past seven years were also analyzed, and there were 125, 168, 156, 164, 158, 209 and 278 cases respectively, accounting for 2.17, 2.83, 2.19, 1.87, 1.48, 1.59, and 1.76% of the total cases. Only 1.91% of the patients could not complete the PFT. The results showed that PFT was a highly successful clinical examination.

The causes of failure were two main types: (1) patients were unable to understand the process of PFT. Majority of them had low educational levels, or in deafness/ blindness condition. (2) Patients did understand the procedure but the test process couldn’t meet the quality control standard. The main conditions included: hesitation at the beginning of blowing, insufficient explosive force, interruption (cough, air leakage) during forced expiration; forced expiration time was less than 6 s, and no end-expiratory platform.

### The incidence of adverse events was only 0.44%

The adverse events were analyzed, the main events were dizziness, amaurosis, limb numbness, lip numbness, and falling down. There were 28, 25, 29, 34, 47, 63 and 71 adverse events respectively in the past seven years, accounting for 0.49, 0.42, 0.41, 0.39, 0.44, 0.48, 0.45% of the total cases. The average incidence of adverse events in the past seven years was about 0.44%, showing that PFT was a very safe clinical examination.

## Discussion

The number of PFT increased from 5754 in 2012 to 15,825 in 2018, and the number of full-time technicians increased from 1 to 4. The number of pulmonary function instruments increased from 1 to 3, in addition, 1 exercise cardiopulmonary function instrument and 1 portable pulmonary function instrument had been equipped. In the past seven years, the proportion of PFT increased from 29.0/ 10,000 in 2012 to 34.7/ 10,000 in 2018. It can be observed that PFT is gradually becoming more and more popular. Through analysis we found the optimized allocation of PFT was 20–25 cases/ person/ day, but no similar research was found so it might need further discussion. According to the monthly changes of PFT in the past seven years, around March and November every year was relatively peak time for this test, suggesting more workload would be needed during these two months. Also, to optimize the quantity and quality of PFT, the staff need to have training regularly [8, 9].

The revenue of PFT in our hospital in 2018 was about 3.7 million Chinese Yuan, significantly higher than the expenditure 800,000 Chinese Yuan. If every hospital allocated the full-time staff and equipment in the PFT room reasonably, the PFT still had certain economic benefits if the hospital had sufficient patients. In the past seven years, 58.2% of the patients who had undergone PFT had various degrees of ventilatory dysfunction, which further suggested that PFT could help detecting early primary or secondary respiratory diseases, so that these patients could be treated and managed early. These data showed that PFT not only have certain economic benefits, but also have significant clinical value in the early diagnosis and early intervention of the disease [10, 11].

The outpatients took PFT mainly came from PCCM, accounting for 97.2, 97.1, 97.3, 97.8, 97.8, 98.0, and 98.2% of the total outpatient cases in the past seven years. In addition, 22 patients from DPE each year added PFT to their routine test, indicating the people began to understand the importance of PFT, also indicating the vital role of PFT in the diagnosis and treatment of respiratory diseases [12–14]. The inpatients took PFT mainly came from DTG, DGS and DUS, accounting for 65.1, 64.4, 62.1, 63.5, 62.4, 65.3 and 69.1% of the total cases in the same period. The PFT was mainly carried out before the operation, which also showed the important role of PFT in the risk assessment of operation especially thoracic surgery or in patients suffering from a respiratory disease. The distribution of PFT in inpatient and outpatient of our hospital showed that PFT has been widely used in various clinical departments. The application of PFT in surgical departments revealed the importance of PFT in surgical risk assessment of patients undergoing thoracoabdominal surgery [3]. More evidence-based medicine is needed to determine whether every patient undergoing chest and or abdominal surgery should be prescribed PFT.

In the past seven years, 56.7, 57.9, 63.8, 58.3, 60.0, 57.8, and 55.1% of the people over 60 years old had undergone PFT, among which 6.7, 7.2, 8.8, 7.0, 6.5, 6.8 and 5.7% were over 80s. Although the vast majority of patients were elderly patients, but the rate of failure to complete PFT was still very low. During the last seven years, the failure rate of PFT was only 1.91%. All data suggested that older age is no longer a limiting factor for PFT, and older people can also routinely perform PFT. Studies had shown that there was no significant difference in the quality of PFT between older adults (> 80 years old) and younger adults (40–50 years old) [15]. These findings were consistent with our findings. The two main causes of failure of PFT were as follows: (1) unable to understand the procedure of PFT, due to low education, hearing loss or blindness; (2) test procedure couldn’t meet the quality control standard [16]. The results suggested that the demonstration and communication before PFT should be further strengthened for the special population with low educational level, deafness and blindness; recommending the supine position for patients with spinal cord injury and neuromuscular disease [17], which might further reduce the failure rate of PFT.

The average incidence of adverse events during PFT in the last seven years was only 0.44%, mainly related to hyperventilation during PFT. Although the incidence of adverse events was slightly higher than another study [18], these were not severe ones, including: dizziness, amaurosis, limb numbness, lip numbness and falling down. It suggested that PFT is still a very easy and safe diagnostic test [19, 20]. As long as indications and contraindications of PFT were well followed, and demonstration and communication with patients was well done, the test could be done for elderly patients easily too [15, 21].

### Study limitations

The main limitation of this study was that the results of single-center data analysis did not necessarily represent the situation of other hospitals. The study was a retrospective analysis, the failure rate of the PFT couldn’t be analysed by multivariate regression. In future, multi-center data will be conducted, which might bring more experience to promote the popularization of PFT.

## Conclusions

In summary, by analyzing the data of PFT in our hospital in the past seven years, we found that the number of PFT is increasing year by year, and the best allocation of staffing is 20–25 cases/person/day. The failure rate and incidence of adverse events in PFT were very low, showing the test is safe and practical. In the future, more attention should be paid to the standardization and generalization of PFT.

## Data Availability

The datasets used and/or analysed during the current study are available from the corresponding author on reasonable request.

## Abbreviations

DCM: Department of Cardiovascular Medicine
DCS: Department of Cardiovascular Surgery
DG: Department of Gastroenterology
DGP: Department of General Practice
DGS: Department of General Surgery
DH: Department of Hematology
DN: Department of Neurosurgery
DOG: Department of Obstetrics and Gynaecology
DOr: Department of Orthopedics
DOt: Department of Otorhinolaryngology
DTS: Department of Thoracic Surgery
DUS: Department of Urinary Surgery
PCCM: Department of Pulmonary and Critical Care Medicine
PFT: Pulmonary function tests
